# Enzymatic Synthesis of Amoxicillin in a Batch Reactor: Mathematical Modeling, Sensitivity Analysis, and Experimental Validation

**DOI:** 10.1021/acsomega.5c03288

**Published:** 2025-07-18

**Authors:** Artur Pedro Martins Neto, Ana Luiza Souza Tavares, Lucas Figueiredo Formigosa, Bruno Duarte Gomes, Luciana Rocha Barros Gonçalves, Bruno Marques Viegas

**Affiliations:** † Faculty of Biotechnology, 37871 Federal University of Pará, Belém, PA 66075-110, Brazil; ‡ Laboratory of Neurophysiology Eduardo Oswaldo Cruz, Institute of Biological Science, Federal University of Pará, Belém, PA 66075-110, Brazil; § Simulation and Computational Biology Laboratory, High Performance Computing Center, Federal University of Pará, Belém, PA 66075-110, Brazil; ∥ Department of Chemical Engineering, 28121 Federal University of Ceará, Fortaleza, CE 60455-760, Brazil; ⊥ Graduate Program in Biotechnology, Federal University of Pará, Belém, PA 66075-110, Brazil

## Abstract

This study evaluated two kinetic models for the enzymatic synthesis of amoxicillin catalyzed by penicillin G acylase, using the Markov chain Monte Carlo (MCMC) method for estimating the process’s kinetic parameters. The first model, based on Michaelis–Menten kinetics, and the second, founded on reaction and equilibrium constants, were optimized using a single initial condition and subsequently validated under 12 distinct experimental conditions. Sensitivity analysis enabled the identification of the most sensitive parameters for each model, while the selection of the model that best fits the experimental measurements was based on Bayesian metrics and the relative mean squared error. The model based on reaction and equilibrium constants demonstrated superior predictive capability, exhibiting a 18.40% error after optimization compared to the 25.79% observed in the Michaelis–Menten model. These results underscore the efficacy of integrating mathematical modeling, Bayesian statistics, and sensitivity analysis in predicting amoxicillin production under different experimental conditions.

## Graphical abstract

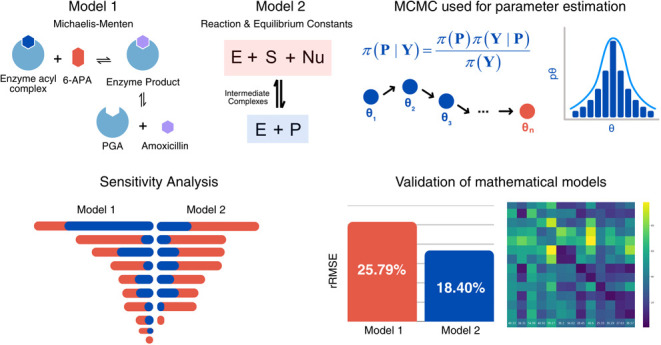

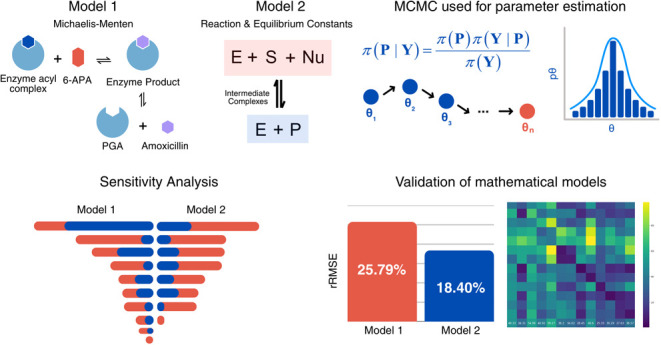

## 1 Introduction

Amoxicillin is a semisynthetic antibiotic of the penicillin class, widely used in the treatment of bacterial infections. Its therapeutic efficacy is attributed to its stability in acidic environments, high gastrointestinal bioavailability, potent antimicrobial activity, and low toxicity.^1^ The primary mechanism of action of this antibiotic involves inhibiting bacterial cell wall synthesis, leading to cell lysis and the subsequent elimination of susceptible microorganisms that rely on the structural integrity of this wall for survival.^2^

Conventional amoxicillin synthesis is based on chemical processes involving reactive groups and toxic solvents, leading to challenges such as the use of hazardous reagents and the requirement for multiple reaction steps.^3^ Enzymatic synthesis emerges as a sustainable alternative by eliminating the use of toxic solvents commonly required in chemical processes. Penicillin G acylase (PGA), identified by Sakaguchi and Murao,^4^ catalyzes the hydrolysis of penicillin G to form 6-aminopenicillanic acid (6-APA).

PGA catalyzes amoxicillin through the reaction between a β-lactam nucleus and an activated acyl donor. In this process, 6-APA acts as the nucleophile, while *p*-hydroxyphenylglycine methyl ester (POHPGME) functions as the activated acyl donor. Under kinetic control, PGA also catalyzes undesirable hydrolysis reactions, leading to the degradation of POHPGME (primary hydrolysis) and the antibiotic product (secondary hydrolysis).^3,5,6^

The development of accurate mathematical models to represent complex enzymatic reactions provides important information about the principles of process optimization and control.^7^ Gonçalves et al.^8^ and McDonald et al.^9^ proposed two distinct kinetic models, based on fundamental principles, to describe PGA-catalyzed amoxicillin synthesis. These modeling approaches enable performance prediction and improvement in enzymatic synthesis, contributing to more efficient and scalable production methods.

Although a comprehensive mechanistic model that includes all possible reaction steps and inhibitory interactions could theoretically describe the system under various operational conditions, this approach often leads to overparameterization and becomes computationally challenging. As noted by Gonçalves et al.,^10^ creating such complex models can result in structures that are impractical for parameter estimation, model validation, or process optimization—especially when experimental data is limited or when many parameters are highly correlated. Therefore, in this work, the motivation for employing these models came from their complementary characteristics. The model proposed by Gonçalves et al.^8^ stands out for its simplicity and ease of parametrization, while the mechanistic equilibrium-based model described by McDonald et al.^9^ offers a more detailed representation of the enzymatic process. Evaluating and validating these two approaches under the same experimental conditions allows for a critical assessment of their predictive capabilities, practical applicability, and suitability for process optimization. Given the increasing industrial demand for β-lactam antibiotics and the necessity for sustainable and efficient enzymatic processes, it is crucial to develop modeling frameworks that achieve a balance between accuracy and computational feasibility.

Within the Bayesian framework, the Markov chain Monte Carlo (MCMC) method is a robust approach for parameter estimation, enabling comprehensive exploration of the parameter space and generating posterior distributions that quantify estimation uncertainty.^11–15^ This approach integrates principles from Monte Carlo methods, which estimate distributions through random sampling, with Markov chain techniques, where each sample depends only on the previous one.^16–18^

This study validates and compares two kinetic models for enzymatic amoxicillin synthesis in a batch reactor, identifying the most sensitive parameters, estimating them from a single initial condition, followed by validation in different experimental conditions. By using the MCMC method, the application of Bayesian methods to enzymatic processes is enhanced, incorporating prior information and contributing to the development of more robust models for enzymatic synthesis.

The novelty of this work lies in the integration of advanced Bayesian statistical techniques, specifically MCMC, with deterministic kinetic modeling of enzymatic synthesis. This integration is followed by a systematic comparison of two established kinetic models using a unified methodological framework for parameter estimation, validation, and sensitivity analysis. This approach identifies key sensitive parameters through Sobol sensitivity analysis, offering new insights into model structure and the influence of parameters. By employing this comprehensive approach, the system behavior can be accurately predicted across a wide range of conditions, which contributes to the design of more efficient and scalable processes for amoxicillin synthesis.

## 2 Materials and Methods

### 2.1 Experimental Measurements

The experimental measures used in this study were obtained from Gonçalves et al.,^19^ consisting of 13 batch experiments conducted at 25 °C and pH 6.5 in a 50 mL batch reactor under mechanical stirring. Initial concentrations of POHPGME (5–100 mM) and 6-APA (30–80 mM) were systematically varied, as shown in Table 1.

**Table 1:** Initial Concentrations of POHPGME and 6-APA for PGA-catalyzed Amoxicillin Synthesis

experiment	6-APA (mM)	POHPGME (mM)
1	30	30
2	80	5
3	80	40
4	40.5	21.75
5	30.4	43
6	40	100
7	55	55
8	55	5
9	55	10
10	32.5	78
11	80	20
12	38.75	12
13	30	5

### 2.2 Mathematical Modeling

#### 2.2.1 Model 1

In this study, two distinct kinetic models were evaluated to describe the enzymatic synthesis of amoxicillin by the enzyme PGA. Model 1, proposed by Gonçalves et al.,^8^ is based on Michaelis–Menten kinetics and focuses on three main processes: amoxicillin synthesis, ester hydrolysis, and amoxicillin hydrolysis, as seen in Figure 1. To represent these processes, based on the proposed mechanism and the reaction stoichiometry, the Michaelis–Menten-based kinetic model describes three key reaction rates: ester consumption (*v*_AB_), amoxicillin hydrolysis (*v*_h2_), and amoxicillin synthesis (*v*_s_), as represented by eqs 1–3.

**Figure 1:**
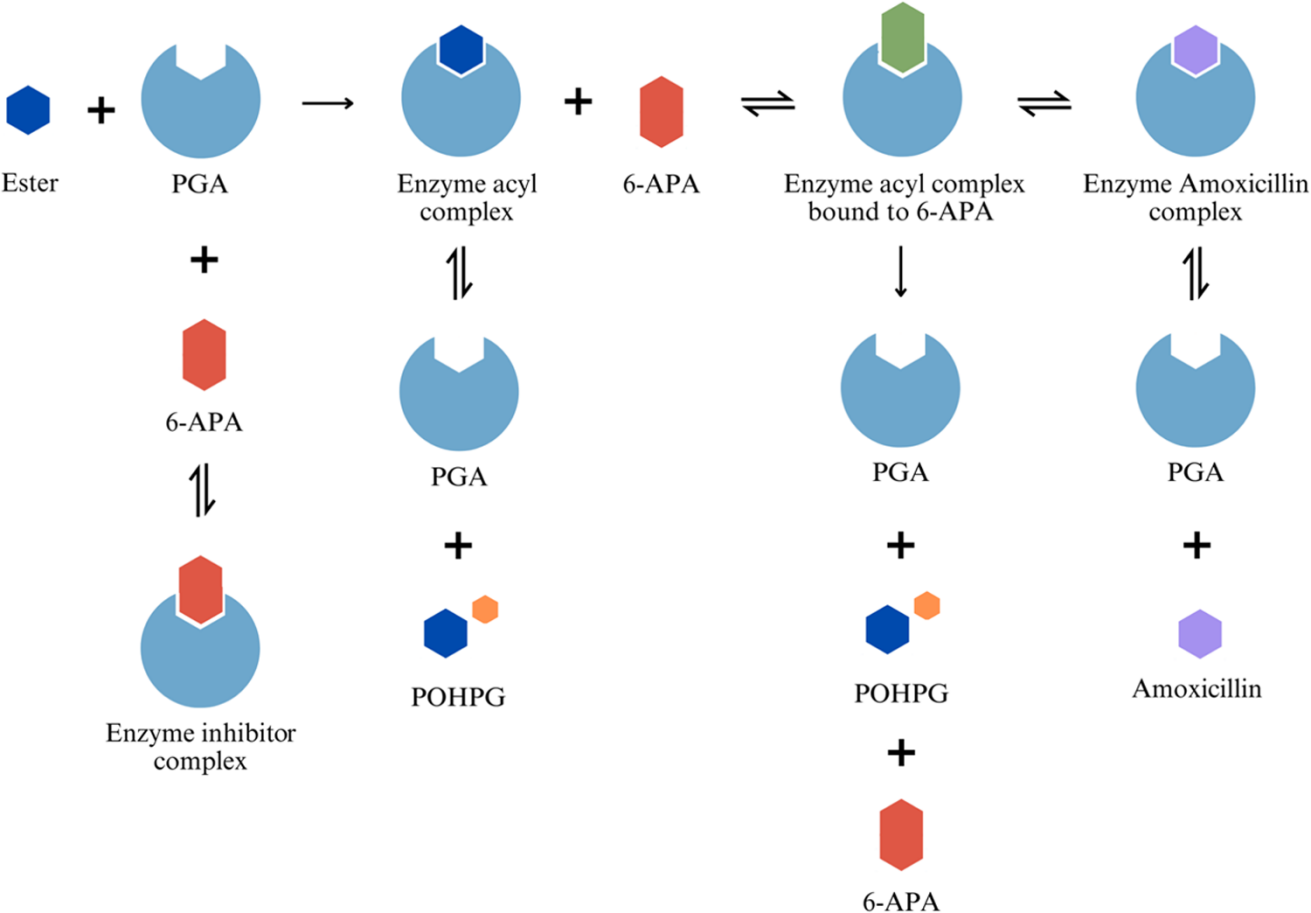
Mechanistic pathway for PGA-catalyzed amoxicillin synthesis (Model 1).

This Michaelis–Menten-based kinetic model describes three key reaction rates: ester consumption (*v*_AB_), amoxicillin hydrolysis (*v*_h2_), and synthesis (*v*_s_), as represented by eqs 1–3.

1 $$v_{\mathrm{AB}}=\frac{k_{\mathrm{cat}1}C_{E}C_{\mathrm{AB}}}{K_{m1}\left(1+\frac{C_{\mathrm{AN}}}{k_{\mathrm{AN}}}+\frac{C_{\mathrm{NH}}}{k_{\mathrm{AOH}}}+\frac{C_{\mathrm{AOH}}}{k_{\mathrm{AOH}}}\right)+C_{\mathrm{AB}}}$$

2 $$v_{h2}=\frac{k_{\mathrm{cat}2}C_{E}C_{\mathrm{AN}}}{K_{m2}\left(1+\frac{C_{\mathrm{AB}}}{k_{\mathrm{AB}}}+\frac{C_{\mathrm{NH}}}{k_{\mathrm{NH}}}+\frac{C_{\mathrm{AOH}}}{k_{\mathrm{AOH}}}\right)+C_{\mathrm{AN}}}$$

3 $$v_{S}=\frac{v_{\mathrm{AB}}C_{\mathrm{NH}}T_{\max}}{K_{\mathrm{EN}}+C_{\mathrm{NH}}}$$

in which *C*_AB_, *C*_E_, *C*_AN_, *C*_NH_ and *C*_AOH_ are the concentrations of POHPGME, PGA, amoxicillin, 6-APA and POHPG, respectively. The model incorporates first-order catalytic rate constants, *K*_cat1_ and *K*_cat2_, which characterizes ester consumption and amoxicillin hydrolysis, respectively. The model also considers several competitive inhibition mechanisms, including: (i) POHPG inhibition in the hydrolysis of POHPGME and amoxicillin (*k*_AOH_); (ii) 6-APA as a competitive inhibitor in amoxicillin hydrolysis (*k*_NH_); (iii) ester as a competitive inhibitor of amoxicillin hydrolysis (*k*_AB_); and (iv) amoxicillin as a competitive inhibitor of ester hydrolysis (*k*_AN_). Additionally, the model incorporates the adsorption of 6-APA to the enzyme (*K*_EN_) and considers the maximum enzymatic conversion (*T*_MAX_) to achieve maximum enzyme activity during the reaction.

In this study, the time-dependent behavior of substrate and product concentrations, as described by the proposed Model 1, is governed by a system of ordinary differential equations (ODEs) presented in eqs 4–7. These equations are subject to mass conservation constraints for each chemical species, ensuring that the total mass of reactants and products remains consistent throughout the reaction process.

4 $$\frac{dC_{\mathrm{AN}}}{dt}=v_{S}-v_{h2}$$

5 $$\frac{dC_{\mathrm{POHPG}}}{dt}=\left(v_{\mathrm{AB}}-v_{S}\right)+v_{h2}$$

6 $$\frac{dC_{6‐\mathrm{APA}}}{dt}=-\left(v_{S}-v_{h2}\right)$$

7 $$\frac{dC_{\mathrm{POHPGME}}}{dt}=-v_{\mathrm{AB}}$$

#### 2.2.2 Model 2

Model 2, based on the work of McDonald et al.,^9^ offers an alternative approach to describing the enzymatic process. This model relies on a set of ODEs that depend on various conversion and equilibrium constants specific to the PGA enzymatic process, including *K*_S_, *K*_P_, *K*_N_, *k*_2_, *k*_3_, *k*_4_, *k*_‑4_, and *k*_5_. This equilibrium-based model describes the reaction system through concentration changes based on elementary reaction steps, as illustrated in Figure 2 and represented by eqs 8–11.

**Figure 2:**
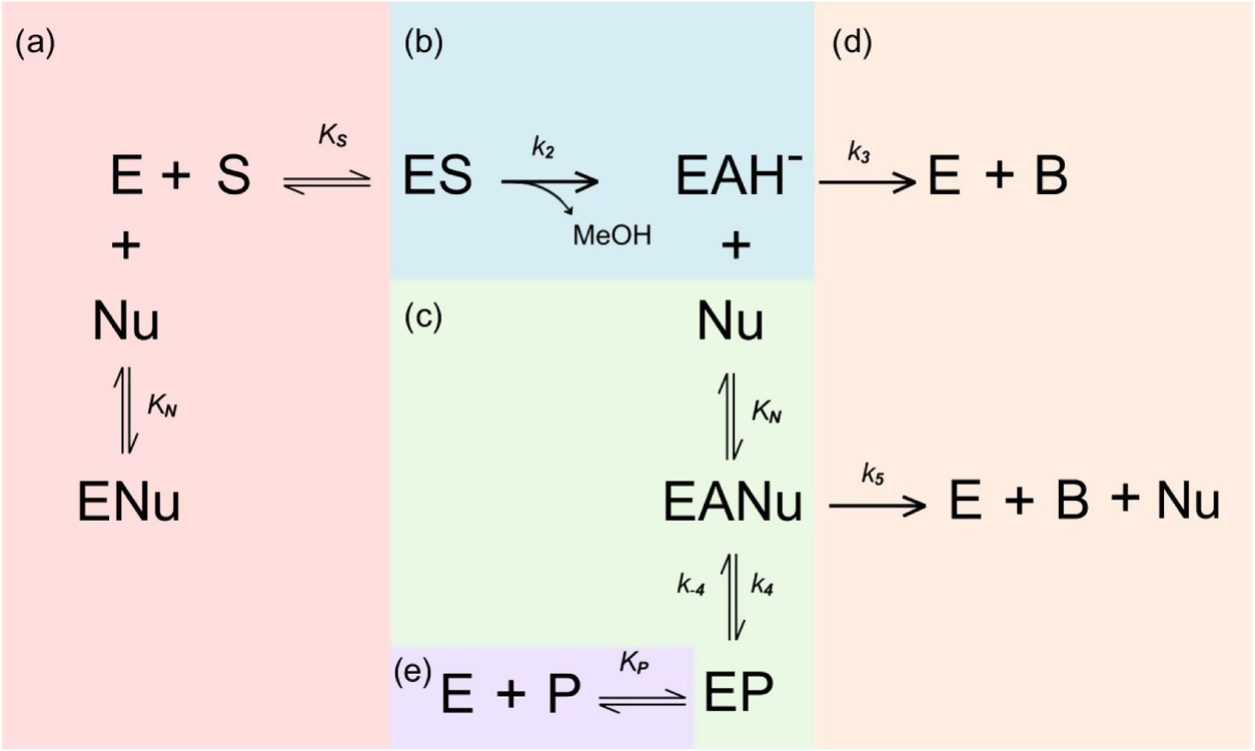
Mechanistic pathway for PGA-catalyzed amoxicillin synthesis (Model 2). Reaction steps: (a) substrate binding to enzyme; (b) acyl-enzyme (EAH) formation; (c) nucleophile attack yielding acyl-enzyme intermediate (EANu); (d) competing hydrolysis reactions forming byproduct POHPG; (e) product release regenerating free enzyme. Rate or equilibrium constants (*K*_S_, *k*_2_, *K*_N_, *k*_3_, *k*_4_, *k*_5_, *k*_–4_ and *K*_P_) are shown on the corresponding arrows.

8 $$\frac{dC_{\mathrm{AN}}}{dt}=\frac{C_{E}}{k_{3}K_{N}+\left(k_{4}+k_{5}\right)C_{\mathrm{NH}}}\left(\frac{k_{2}k_{4}C_{\mathrm{AB}}C_{\mathrm{NH}}}{K_{S}}-\frac{k_{-4}C_{\mathrm{AN}}\left(k_{3}K_{N}+k_{5}C_{\mathrm{NH}}\right)}{K_{P}}\right)$$

9 $$\frac{dC_{\mathrm{POHPG}}}{dt}=\frac{C_{E}\left(k_{3}K_{N}+k_{5}C_{\mathrm{NH}}\right)}{k_{3}K_{N}+\left(k_{4}+k_{5}\right)C_{\mathrm{NH}}}\left(\frac{k_{2}C_{\mathrm{AB}}}{K_{S}}-\frac{k_{-4}C_{\mathrm{AN}}}{K_{P}}\right)$$

10 $$\frac{dC_{6‐\mathrm{APA}}}{dt}=-\frac{dC_{\mathrm{AN}}}{dt}$$

11 $$\frac{dC_{\mathrm{POHPGME}}}{dt}=-\left(\frac{dC_{\mathrm{AN}}}{dt}+\frac{dC_{\mathrm{POHPG}}}{dt}\right)$$

The mechanism illustrated in Figure 2 involves: (a) the formation of the enzyme–substrate complex (ES) through the binding of the substrate (POHPGME) to the free enzyme (E), represented by the equilibrium constant *K*_s_; (b) the conversion of the ES complex into the activated acyl-enzyme complex (EAH), characterized by the rate constant *k*_2_; (c) the interaction of the acyl-enzyme complex with the nucleophile (6-APA), forming an enzyme-nucleophile intermediate (EANu), defined by the equilibrium constant *K*_N_; (d) the generation of byproducts (POHPG) and the formation of the antibiotic product (P); and (e) the equilibrium between the intermediate complex and the enzyme–product complex (EP), described by the rate constants *k*_4_ and *k*_*–*4_, which results in the release of the product and the regeneration of the free enzyme (E).^9^

### 2.3 Computational Simulation

The numerical solution of the system of ordinary differential equations was implemented in Python using the BDF (Backward Differentiation Formula) solver from SciPy.^20^ This implicit multistep method, with variable orders ranging from 1 to 5, was applied to accurately model the system’s dynamics, particularly for stiff problems. The solver utilizes a quasi-constant step size scheme and is enhanced with the NDF modification to improve accuracy. The integration process was controlled by relative and absolute tolerance values of 10^–3^ and 10^–6^, respectively, ensuring that the solution maintained the desired precision throughout the simulation. These tolerances were set to balance computational efficiency and error control, with adjustments made when necessary to account for components with varying scales.

### 2.4 Sensitivity Analysis

Before estimating the parameters, a Sobol sensitivity analysis, a global variance-based approach, was conducted to identify the most influential parameters affecting model outputs across all initial conditions. The analysis utilized first-order (*S*_1_) and total-order (*S*_T_) Sobol indices to quantify the contributions of each parameter. Specifically, *S*_1_ indices measured the direct impact of individual parameters, whereas *S*_T_ indices accounted for both direct effects and interactions with other parameters. Consequently, this filtering strategy streamlined the parameter estimation process by prioritizing the most critical variables, thereby reducing model complexity and minimizing calibration uncertainties.^21,22^

Parameter sets for the sensitivity analysis were generated using Saltelli’s extension of the Sobol Sequence, a robust and efficient strategy for variance-based sensitivity analysis.^23^ Using a base sample size of 2048, 44,056 parameter sets were sampled for Model 1, while for Model 2, 36,864 parameter sets were generated. Parameters were sampled within a range of ±260% of their literature-reported values, ensuring a sufficiently broad search space for a robust sensitivity analysis that captures plausible biochemical variability. To quantify the uncertainty in the sensitivity indices, bootstrap resampling (*n* = 1000) was applied to compute 95% confidence intervals for both *S*_1_ and *S*_T_ Sobol indices. This entire analysis was conducted in Python, using SALib v1.5.1 ^24,25^ and NumPy v2.2.6.^26^ This approach ensured the robustness of the analysis and provided a measure of the statistical uncertainty associated with each parameter’s effect.^21^

To represent the time-dependent behavior of the models, sensitivity indices were computed at 201 equally spaced time points over 500 min of the experiment. Analyses were performed under six distinct initial conditions to ensure adequate coverage of the system’s state space and to account for variability. In addition to determining instantaneous sensitivity profiles, the temporal evolution of *S*_1_ and *S*_T_ was assessed by numerically differentiating these indices over time and identifying local maxima or minima in each species’ concentration profile. Mean values of *S*_1_ and *S*_T_ were subsequently calculated over the full 500 min interval to identify parameters exerting the greatest overall influence on model response. Parameters with a mean *S*_T_ value lower than 0.1 were classified as nonsignificant and therefore maintained at the reference values described in the literature. By excluding these low-impact elements, the fitting procedure was concentrated on the most influential parameters, thereby enhancing calibration efficiency and reliability.

### 2.5 Parameter Estimation and Experimental Validation

After the sensitivity analysis and parameter selection, the estimation of these parameters was performed using the MCMC method, as observed in Figure 3. To validate the mathematical models, a cross-validation strategy was implemented, in which the 13 experiments were used to estimate the parameters and assess predictive accuracy across the entire range of concentrations studied. Each experiment yielded a different parameter distribution, which was then used to simulate the remaining 12 experiments, by solving the model from its initial conditions, to evaluate out-of-sample performance. Subsequently, the errors of the models relative to the experimental measurements were calculated and represented in a heatmap. The experiment that exhibited the lowest error was selected for model calibration, while the remaining 12 experiments were used for experimental validation, ensuring a comprehensive and reliable evaluation under various conditions.

**Figure 3:**
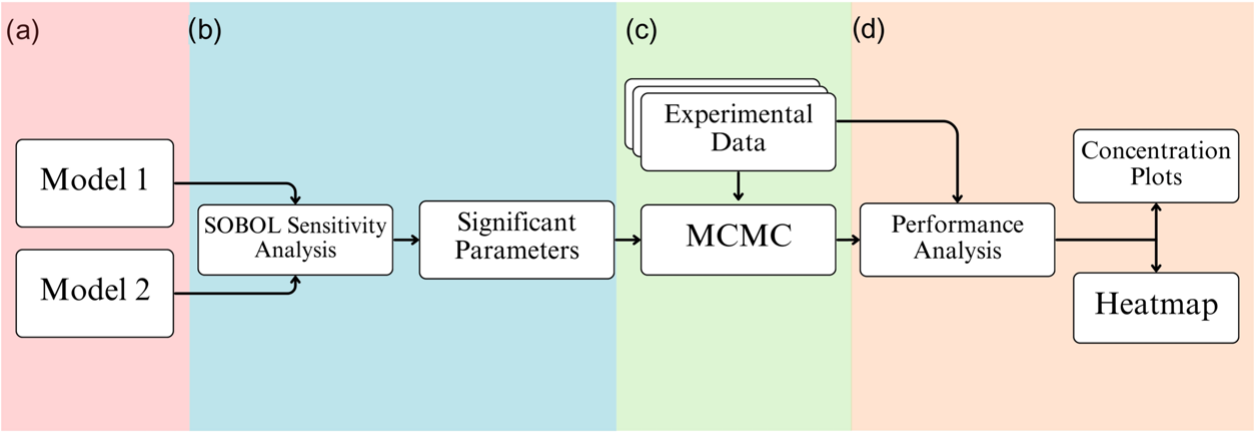
Methodological flowchart: (a) model selection; (b) Sobol sensitivity analysis; (c) parameter estimation via MCMC; and (d) experimental validation.

The MCMC method, based on Bayes’ theorem, combines experimental measurements with an a priori probability distribution to quantify the uncertainties associated with the model parameters. Based on this formulation, the methodology enables determining posterior probability distributions, providing a statistically robust solution for parameter analysis and estimation, as described in eq 12.^27,28^

12 $$\pi \left(P|Y\right)=\frac{\pi \left(P\right)\pi \left(Y|P\right)}{\pi \left(Y\right)}$$

where **P** represents the vector of unknown parameters, and **Y** corresponds to the vector of state variables of the mathematical model. The posterior probability of the parameters, given the observed data, is expressed by π­(**P**|**Y**), while π­(**P**) represents the prior probability distribution. The likelihood function, π­(**Y**|**P**), quantifies the agreement between model predictions and experimental data, and π­(**Y**) is a normalizing constant, ensuring the probabilistic validity of the posterior distribution.^13,14,16^

In this study, two distinct kinetic parameter vectors were estimated: eq 13, corresponding to Model 1, and eq 14, associated with Model 2. The state variables, represented by eq 15, describe the temporal dynamics of the system.

13 $$P_{1}=\left[K_{\mathrm{EN}},K_{m1},K_{m2},T_{\max},k_{\mathrm{AB}},k_{\mathrm{AN}},k_{\mathrm{AOH}},k_{\mathrm{NH}},k_{\mathrm{cat}1},k_{\mathrm{cat}2}\right]$$

14 $$P_{2}=\left[K_{N},K_{P},K_{S},k_{-4},k_{2},k_{3},k_{4},k_{5}\right]$$

15 $$Y=\left[C_{\mathrm{AN}},C_{\mathrm{AB}},C_{\mathrm{NH}},C_{\mathrm{AOH}}\right]$$

The MCMC method was implemented using the Metropolis–Hastings (MH) algorithm, one of the most widely employed in Bayesian statistics. The MH algorithm is a stochastic method used to generate random samples from an arbitrary probability distribution, ensuring the convergence of the Markov chain to the desired equilibrium distribution. In each iteration, a new candidate vector P* is proposed by a probabilistic distribution, typically Gaussian, which generates values close to the current state **P**_curr_, as presented in eq 16.^29,30^

16 $$P^{*}=P_{\mathrm{curr}}+\omega \varepsilon P_{\mathrm{curr}}$$

where ω is the relative standard deviation, defined as 6 × 10^–3^, and ε is a random variable following a Gaussian distribution with zero mean and unit standard deviation. The acceptance or rejection of the candidate is determined by a function that considers both the likelihood and the prior probability distribution, ensuring that the samples correctly follow the parameter space and maintain the statistical consistency of the model.^29,30^

### 2.6 Evaluation Metrics

The precision of the parameter sets estimated for each model was analyzed using the relative root-mean-square error (rRMSE), eq 17, used as a quantitative indicator of the agreement between model predictions and experimental measurements.

17 $$r\mathrm{RMSE}=\sqrt{\frac{\frac{1}{n}\sum_{i=1}^{n}{\left(C_{\exp}-C_{\mathrm{est}}\right)}^{2}}{\max\left(C_{\exp}\right)-\min\left(C_{\exp}\right)}}$$

in which *n* is total number of experimental measurements, max and min are the largest and smallest values of the state variables, respectively, **C**_exp_ and **C**_est_ are the experimental and estimated concentrations, respectively. In addition to rRMSE, the validation of the mathematical models was performed using the Akaike Information Criterion (AIC) and the Bayesian Information Criterion (BIC), with the AIC and BIC calculated for each model and parameter set, whose equations are presented in eqs 18 and 19, respectively.^31^

18 AIC=−2⁡ln⁡(L)+2k

19 BIC=−2⁡ln⁡(L)+k⁡ln⁡(n)

in which *L* represents the likelihood function, and *k* corresponds to the number of estimated parameters in the model.

## 3 Results and Discussion

### 3.1 Sensitivity Analysis Results

The relative influence of model parameters was assessed using Sobol sensitivity analysis, with results presented in Figure 4. The Sobol indices (*S*_1_ and *S*_T_) were calculated for all experimental conditions over time, enabling a comprehensive analysis of parameter sensitivity, considering both direct effects and higher-order interactions. Error bars represent 95% confidence intervals obtained through bootstrap sampling, providing an estimate of the uncertainty associated with the calculated indices.

**Figure 4:**
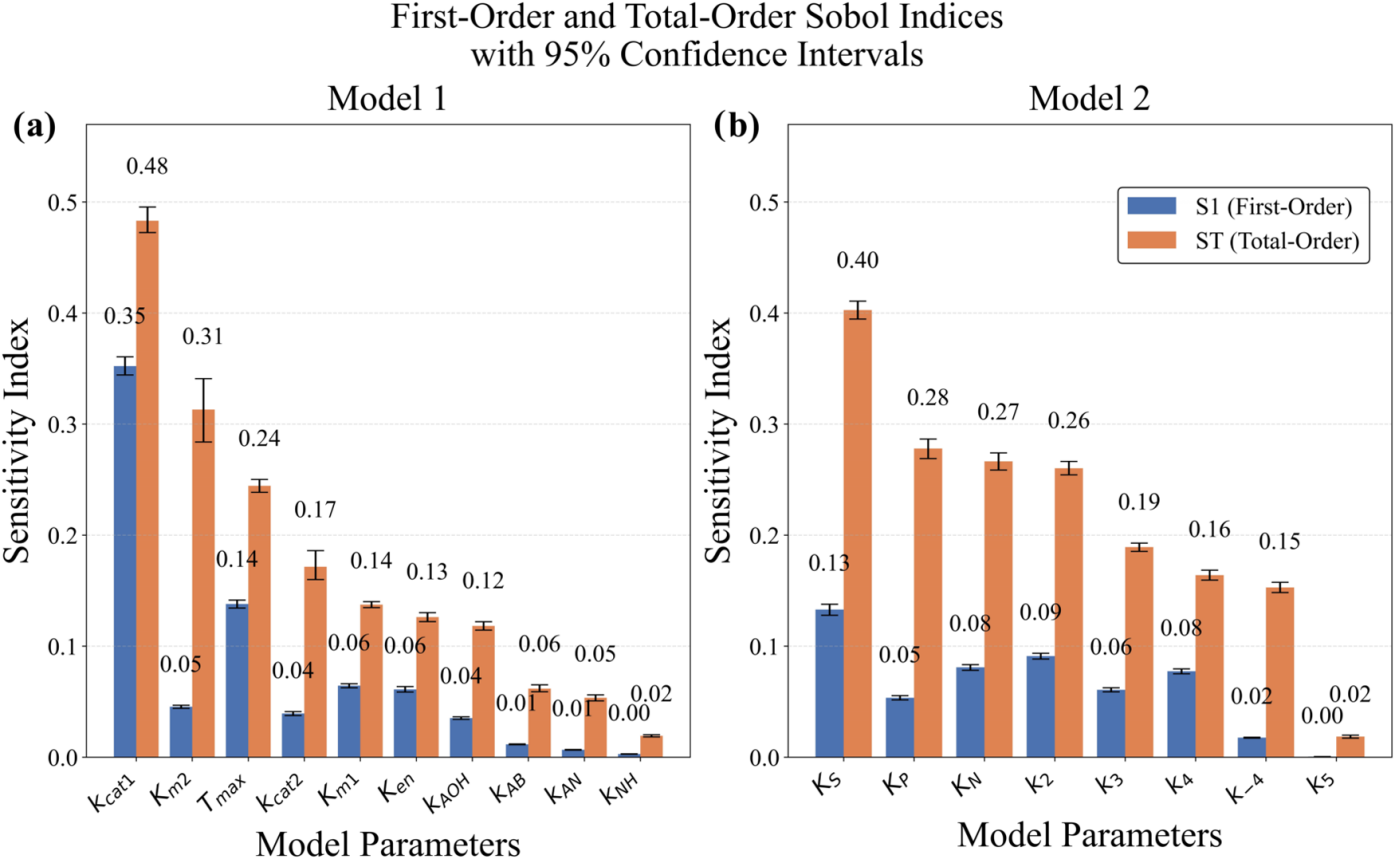
Sobol Sensitivity analysis with 95% confidence intervals for (a) Model 1 and (b) Model 2 parameters.

For Model 1, the analysis revealed different sensitivity values for the parameters. The catalytic rate constant (*K*_cat1_) was identified as the most sensitive parameter, with an *S*_T_ value of 0.48, followed by the Michaelis constant *K*_m2_ (*S*_T_ = 0.31). These total-order indices indicate both significant direct effects and relevant interactions among the parameters that influence the model’s behavior. In contrast, the inhibition constants *k*_AN_, *k*_AB_, and *k*_NH_ exhibited negligible influence, with both *S*_1_ and *S*_T_ values below 0.1, resulting in their exclusion from the estimation process.

In Model 2, the equilibrium constants *K*_S_ and *K*_P_ were the variables with the highest sensitivity, with *S*_T_ indices of 0.40 and 0.28, respectively, highlighting their fundamental role in governing reaction dynamics. On the other hand, the rate constant *k*_5_ exhibited low sensitivity (*S*_1_ < 0.02 and *S*_T_ < 0.05), with minimal influence on the mathematical model, justifying its exclusion from the parameter estimation process.

The systematic sensitivity analysis enabled an optimized reduction of the parameter space by identifying only the most influential parameters for estimation. This refinement not only increased computational efficiency but also strengthened the robustness of the estimation process by prioritizing parameters with a quantifiable influence on model predictions. To quantify the individual contribution of each kinetic parameter to the global response of the system, the temporal gradient of *S*_T_ (d*S*_T_/d*t*) was evaluated for the parameters estimated in Models 1 (Figure 5) and 2 (Figure 6).

**Figure 5:**
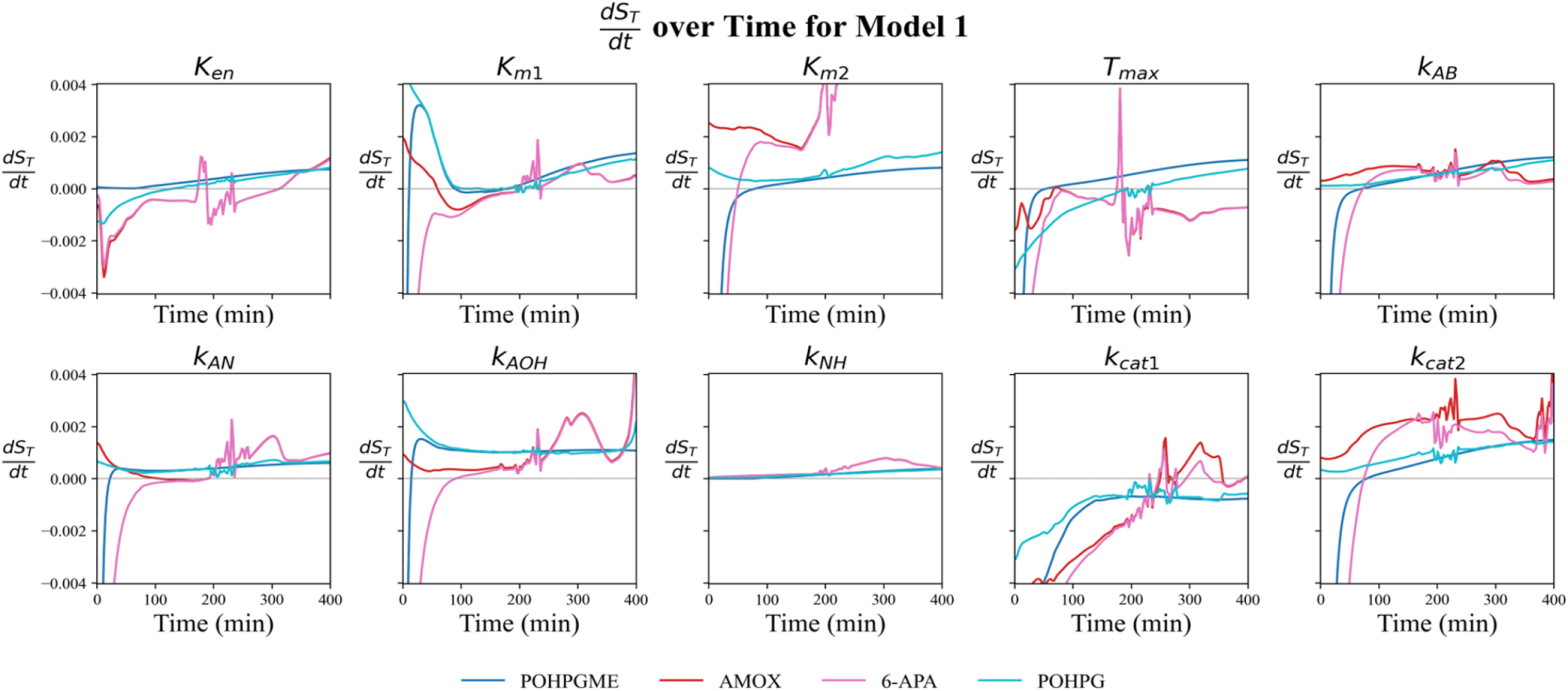
Temporal profiles of d*S*_T_/d*t* for each kinetic parameter of Model 1, obtained as the average across 13 experiments. The curves represent POHPGME (dark blue), amoxicillin (red), 6-APA (magenta), and POHPG (cyan). The gray horizontal line indicates the equilibrium point (d*S*_T_/d*t* = 0).

**Figure 6:**
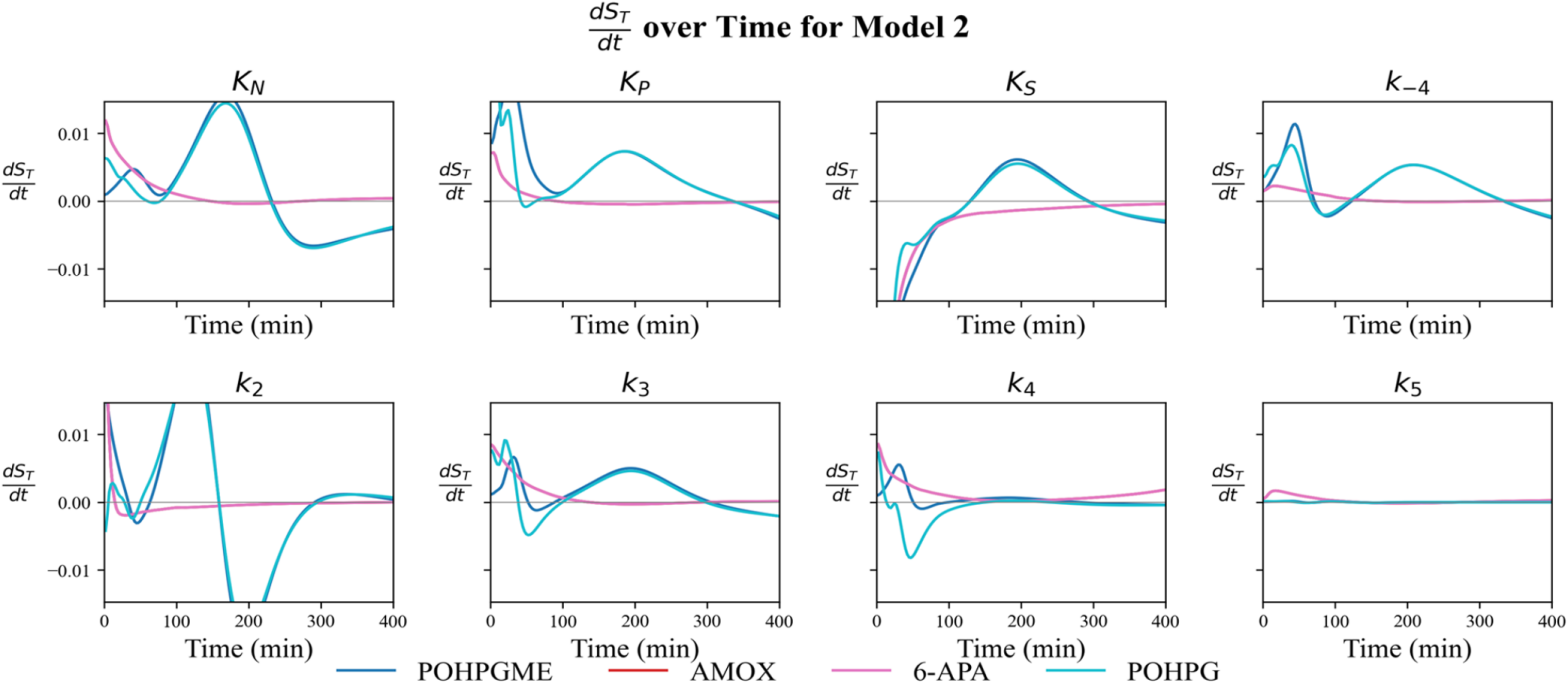
Temporal profiles of d*S*_T_/d*t* for each kinetic parameter of Model 2, obtained as the average across 13 experiments. The curves represent POHPGME (dark blue), amoxicillin (red), 6-APA (magenta), and POHPG (cyan). The gray horizontal line indicates the equilibrium point (d*S*_T_/d*t* = 0).

In Model 1, the time derivative profiles show that the sensitivity indices of most parameters associated with POHPGME and POHPG increase between 50 and 400 min, except for kcat1, in which its influence decreases throughout the interval. For amoxicillin and 6-APA, the indices show similar behaviors, consistent with the opposite stoichiometry of these species in the reaction network. On the other hand, *T*_MAX_ shows a gradual decrease in sensitivity throughout the experiment. The largest variations are in the first 100 min; after this period, most parameters stabilize in a quasi-stationary regime.

As illustrated in Figure 6, the d*S*_T_/d*t* profiles for Model 2 show that the parameter sensitivities for amoxicillin and 6-APA rise until 100 min and then plateau, exhibiting minimal fluctuation thereafter, except for *K*_S_, which decreases continuously during the experiment. For POHPGME and POHPG, the derivatives of *K*_N_, *K*_P_, *K*_*S*_, *k*_–4_, and *k*_3_ reach a maximum value at about 300 min before decreasing, while the sensitivity associated with *k*_2_ peaks at 150 min and then declines to a minimum at the same 300 min time point. Consequently, POHPGME and POHPG exhibit the largest temporal variations in *S*_T_, while amoxicillin and 6-APA exhibit a rapid transition.

The d*S*_T_/d*t* profiles show that the inhibition constants *k*_NH_, *k*_AB_, and *k*_AN_ in Model 1, as well as *k*_5_ in Model 2, have little influence on the system dynamics: their sensitivity indices remain near zero, and the *S*_1_ and d*S*_1_/d*t* analyses confirm these results (see Supporting Information Figures S1–S6). Thus, fixing these parameters simplifies calibration without compromising model validation.

### 3.2 Parameter Estimation and Validation for Model 1

To assess the generalization capacity of Model 1, a detailed heatmap (Figure 7) was generated to represent the rRMSE values. Each element of the heat map corresponds to the performance of estimated parameters for one experiment when applied to different experimental conditions. The cross-validation approach revealed that experiments 3 and 5 provided the best estimates across all tested scenarios. On the other hand, experiments 2 and 8 exhibited high rRMSE values in most parameter estimates, indicating a lower generalization capacity.

**Figure 7:**
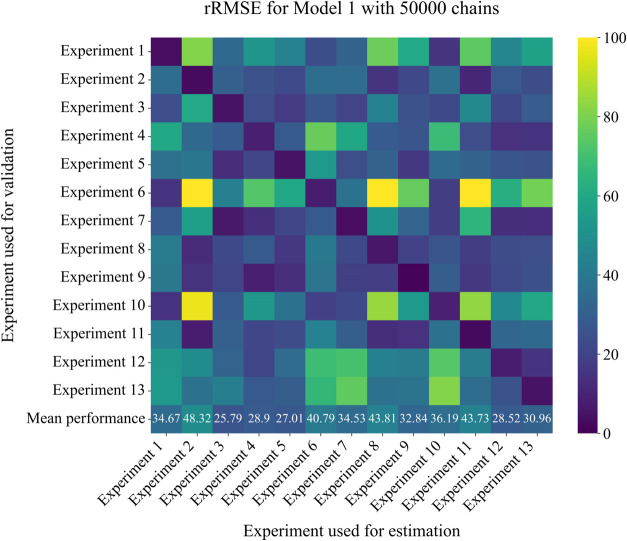
Heat maps for cross-validation of parameter estimation performance for Model 1.

The parameter estimation using MCMC resulted in an improvement in predictive accuracy, with a reduction in mean rRMSE from 27.91 to 25.79%. This improvement indicates that the parameters obtained from the literature were already close to the optimal values for the experimental conditions evaluated.

Figure 8 illustrates the temporal evolution of the concentrations of 6-APA, POHPGME, amoxicillin, and POHPG during experiments 1, 6, 11, and 12. The concentration profiles predicted by Model 1 were compared with the experimental measurements of Gonçalves et al.,^19^ allowing for an assessment of the model’s ability to represent the reaction dynamics. Good agreement is observed between the estimates and the experimental data, validating the mathematical model and indicating that the adopted kinetics satisfactorily describe the formation and consumption rates of these species, even under varying operating conditions (see Supporting Information Figures S7–S9). However, a few minor deviations, particularly after 300 min of experimentation, may be associated with the limitations of the Michaelis–Menten model when characterizing long-duration processes.

**Figure 8:**
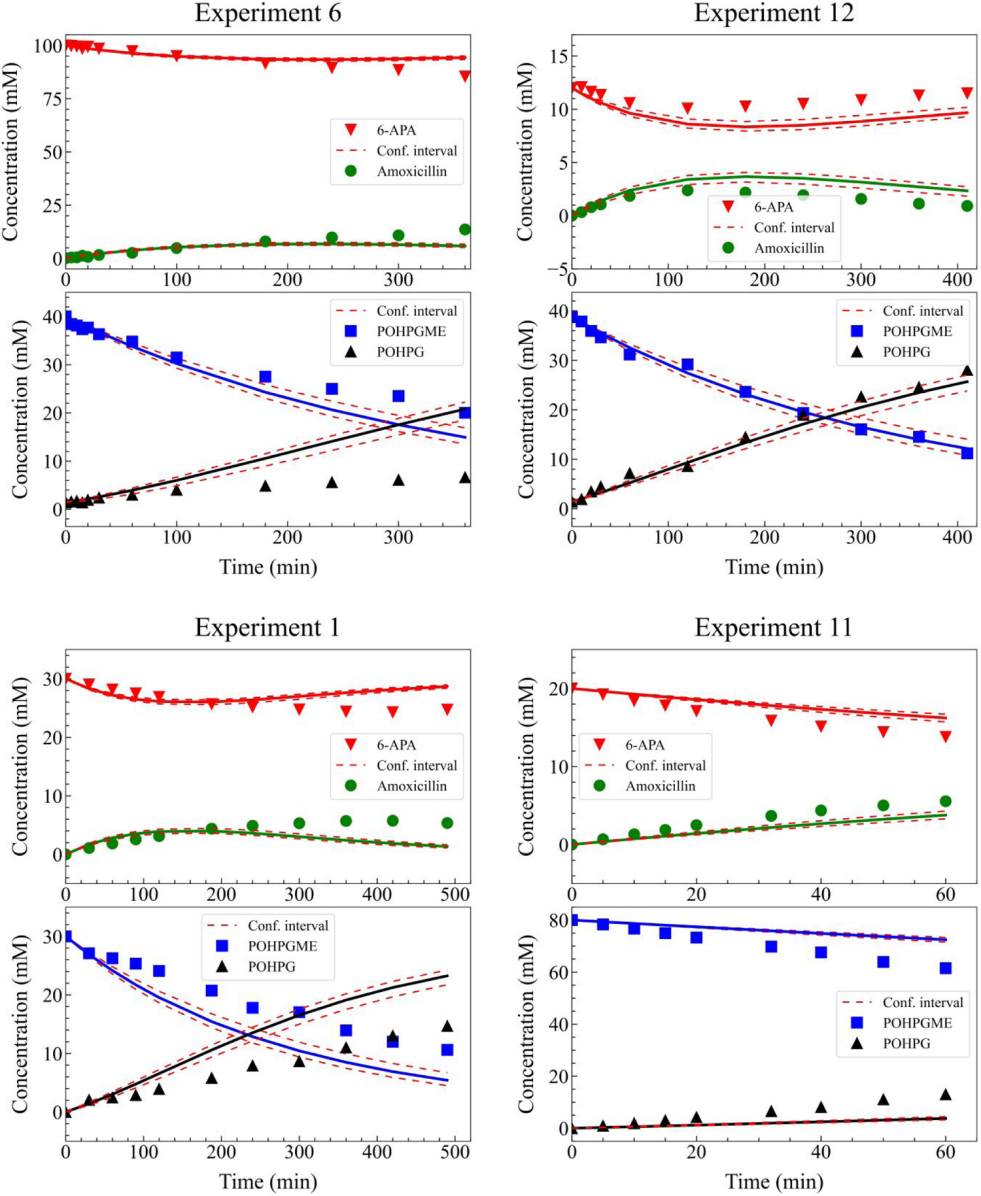
Experimental data and model predictions for the time course concentrations of 6-APA, POHPGME, amoxicillin, and POHPG in four different experiments (experiments 6, 12, 1 and 11) using Model 1. Symbols represent experimental measurements, while solid lines denote model estimates. Shaded areas or dashed lines indicate the 95% confidence intervals for the model predictions.

### 3.3 Parameter Estimation and Validation for Model 2

Cross-validation analysis indicated that Model 2 (Figure 9) was able to represent different initial conditions, exhibiting lower rRMSE values in the heatmap compared to Model 1. This broader applicability suggests that the equilibrium-based approach more effectively captured the fundamental mechanistic aspects of the enzymatic synthesis process, although Model 1 also presents low rRMSE values. The parameter sets derived from experiments 12 and 13 showed reduced rRMSE values when applied to other conditions, thus providing the best estimates in all scenarios evaluated.

**Figure 9:**
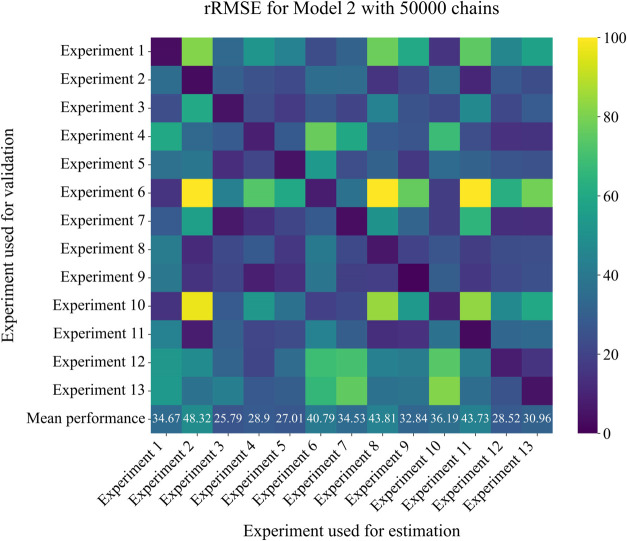
Heat maps for cross-validation of parameter estimation performance for Model 2.

After parameter estimation via MCMC, Model 2 exhibited superior performance compared to Model 1. Using experiments 13 as reference data set, a mean rRMSE of 18.40% was obtained, indicating an improvement over the 25.79% obtained with Model 1. These results highlight the capacity of Model 2 to adequately describe the reaction dynamics.

Figure 10 illustrates the temporal evolution of the concentrations of 6-APA, POHPGME, amoxicillin, and POHPG during experiments 1, 6, 11, and 12. The obtained profiles display good agreement between the experimental measurements and the model-estimated concentrations, with almost all measurements within the confidence interval (see Supporting Information Figures S10–S12).

**Figure 10:**
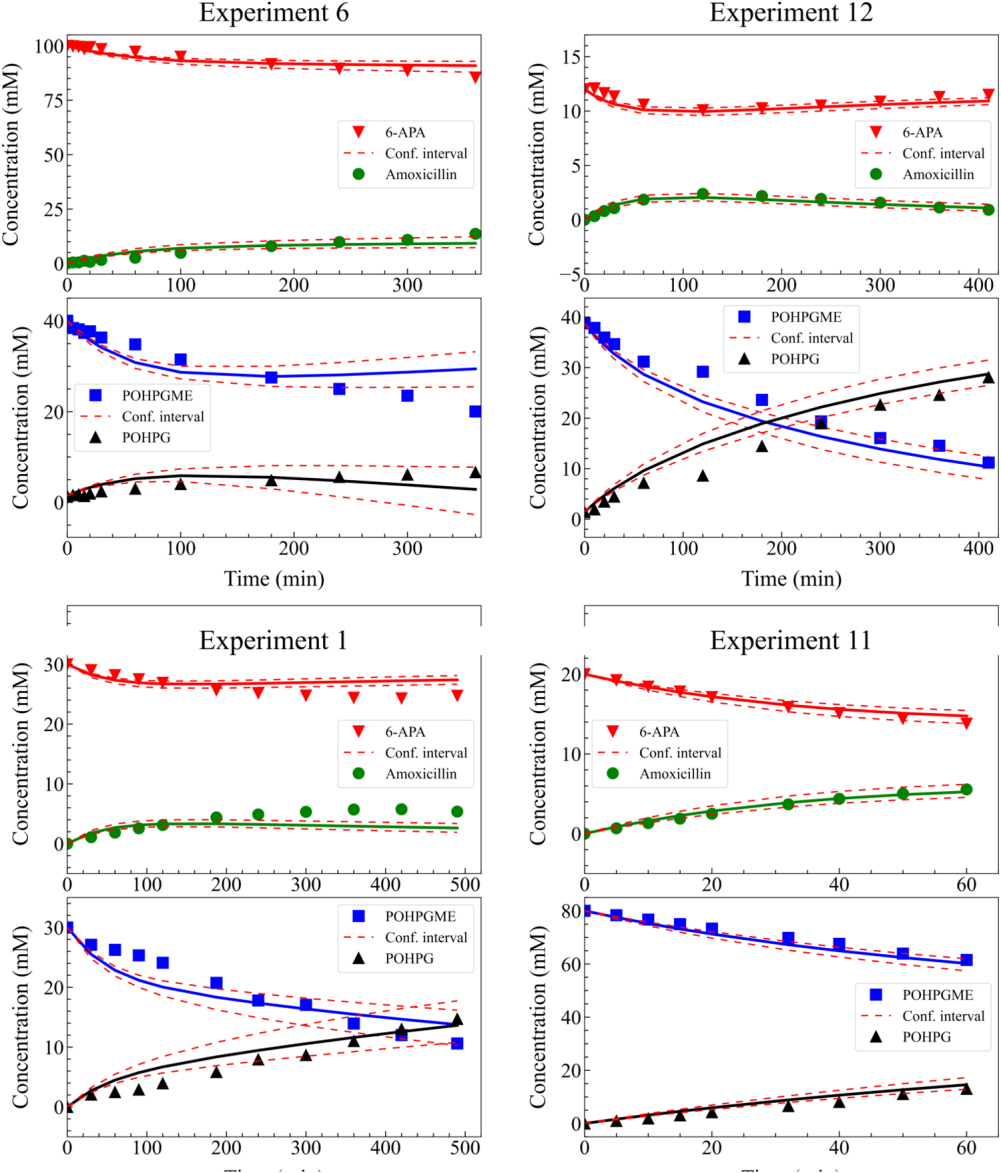
Experimental data and model predictions for the time course concentrations of 6-APA, POHPGME, amoxicillin, and POHPG in four different experiments (experiments 6, 12, 1 and 11) using Model 2. Symbols represent experimental measurements, while solid lines denote model estimates. Shaded areas or dashed lines indicate the 95% confidence intervals for the model predictions.

### 3.4 Optimal Parameters

Tables 2 and 3 present the parameters optimized via MCMC, expressed in terms of the mean and the 95% confidence interval. Parameters marked with (*) were fixed based on literature values due to their low sensitivity. For Model 1, the estimation used experiment 3 as the reference, while for Model 2, the optimization was carried out based on experiment 13. The selection of these experiments was made because of their superior performance in cross-validation and the lowest rRMSE values observed under all experimental conditions.

**Table 2:** Kinetic Parameters of Model 1 Estimated Using the Markov Chain Monte Carlo (MCMC) Method, Compared with Values Previously Reported by Gonçalves et al.^8^ ^,^ ^a^

parameter	Gonçalves et al.^8^	MCMC estimated
*k*_cat1_ (μmol IU^–1^ min^–1^)	0.178	0.153 ± 0.000
*k*_cat2_ (μmol IU^–1^ min^–1^)	0.327	0.198 ± 0.001
*K*_m1_ (mM)	7.905	11.816 ± 0.022
*K*_m2_ (mM)	12.509	2.683 ± 0.014
*T*_max_ (dimensionless)	0.606	0.761 ± 0.001
*K*_en_ (mM)	14.350	6.732 ± 0.038
*k*_AB_ (mM)	3.780	3.780*
*k*_AN_ (mM)	9.174	9.174*
*k*_AOH_ (mM)	10.907	16.153 ± 0.066
*k*_NH_ (mM)	62.044	62.044*

a Parameter estimates are presented with their associated standard deviations (±). Parameters marked with an asterisk (*) were fixed during the MCMC estimation based on literature values and were not re-estimated.

**Table 3:** Kinetic Parameters of Model 1 Estimated Using the Markov Chain Monte Carlo (MCMC) Method, Compared with Values Previously Reported by McDonald et al.^9^ ^,^ ^a^

parameters	McDonald et al.^9^	MCMC estimated
*k*_2_ (min^–1^)	3.117	1.110 ± 0.005
*k*_3_ (min^–1^)	0.733	0.164 ± 0.001
*k*_4_ (min^–1^)	3.917	0.291 ± 0.001
*k*_5_ (min^–1^)	0.150	0.150*
*K*_S __(mM)	380.0	171.443 ± 0.781
*K*_P_ (mM)	95.0	39.167 ± 0.090
*K*_N_ (mM)	43.0	48.485 ± 0.212
*k*_–4_ (min^–1^)	3.617	0.970 ± 0.003

a Parameter estimates are presented with their associated standard deviations (±). Parameters marked with an asterisk (*) were fixed during the MCMC estimation based on literature values and were not re-estimated.

The parameter estimates for Model 1 exhibited minor deviations from those reported by Gonçalves et al.^8^ The main adjustments included a 49.5% reduction in *K*_m2_ (from 12.509 to 2.683 mM) and a 53.1% decrease in *K*_en_ (from 14.35 to 6.732 mM). In contrast, the optimized parameters for Model 2 showed significant differences compared to the literature values, especially regarding the kinetic rate constants, with a 92.57% reduction in k4 (from 3.917 to 0.291 min^–1^) and a 73.18% decrease in *k*_–4_ (from 3.617 to 0.970 min^–1^). The parameter estimates improved the predictive accuracy of the models, as validated by experimental measurements, evidencing the effectiveness of the MCMC method.

### 3.5 Model Performance and Selection

Table 4 presents the comparison of the rRMSE, AIC, and BIC values for Models 1 and 2, using parameters obtained from the literature and estimated via MCMC.^8,9^ The results indicate that the parameters optimized by MCMC exhibit lower rRMSE, AIC, and BIC values in both models, indicating an improvement in predictive capacity.

**Table 4:** Comparison of Model Performance Metrics for Models 1 and 2, Including Relative Root Mean Square Error (rRMSE, %), Akaike Information Criterion (AIC), and Bayesian Information Criterion (BIC)

model	rRMSE (%)	AIC	BIC
model 1 (Gonçalves et al.^8^)	27.91	27.9	71.1
model 1 (MCMC)	25.79	26.1	69.3
model 2 (McDonald et al.^9^)	85.16	84.4	118.9
model 2 (MCMC)	18.40	18.7	53.8

## 4 Conclusions

In this study, mathematical modeling and validation were performed for the enzymatic synthesis of amoxicillin catalyzed by penicillin G acylase, using the MCMC method and Sobol sensitivity analysis. Two kinetic models were considered: Model 1, a widely used Michaelis–Menten-based approach, valued for its simplicity and empirical applicability; and Model 2, a mechanistically detailed model based on reaction and equilibrium constants. This comparison allowed us to systematically evaluate the trade-offs between model complexity and predictive accuracy. Model 2 demonstrated superior performance compared to Model 1, yielding rRMSE values below 20% and showing greater versatility at different initial concentrations of POHPGME and 6-APA.

The integration of mathematical validation with sensitivity analysis enabled the identification of the most sensitive parameters for each approach, highlighting *K*_cat1_ and *K*_m2_ in Model 1 and *K*_S_ and *K*_P_ in Model 2. The application of the MCMC method, combined with cross-validation, improved the estimates of kinetic parameters and the models’ precision, enabling the prediction of a wide variety of experimental conditions without the need for additional assays.

This study improved the mathematical modeling of the enzymatic synthesis of amoxicillin by integrating Bayesian statistics with sensitivity analysis, providing a consistent framework for parameter estimation, uncertainty quantification, and experimental validation. Beyond amoxicillin, the same methodology can be applied to other products obtained through enzymatic catalysis, reducing the need for additional experiments and accelerating process optimization. Future investigations should explore alternative kinetic models and validate the approach at pilot or industrial scales, thereby expanding the impact of this strategy on the advancement of pharmaceutical synthesis processes.

## Supplementary Material



## Notes

Time-course of total-order Sobol indices, *S*_T_, for Model 1 (Figure S1) and Model 2 (Figure S2); time-course of first-order Sobol indices, *S*_1_, for Model 1 (Figure S3) and Model 2 (Figure S4); temporal derivatives of *S*_T_ for Model 1 (Figure S5) and Model 2 (Figure S6); experimental versus model-predicted concentrations of 6-APA, POHPGME, amoxicillin and POHPG for Model 1 validation (Figures S7–S9) and Model 2 validation (Figures S10–S12) (PDF)

## Nomenclature and Symbols

**Nomenclature**
6-APA: 6-aminopenicillanic acid
AIC: Akaike Information Criterion
BDF: backward differentiation formula
BIC: Bayesian Information Criterion
PGA: penicillin G acylase
MCMC: Markov chain Monte Carlo
MH: Metropolis–Hastings
ODE: ordinary differential equation
POHPG: *p*-hydroxyphenylglycine
POHPGME: *p*-hydroxyphenylglycine methyl ester
rRMSE: relative root-mean-square error
**Symbols**
*C*_AB_: concentration of POHPGME (mM)
*C*_AN_: concentration of amoxicillin (mM)
*C*_AOH_: concentration of POHPG (mM)
*C*_E_: concentration of free enzyme (PGA) (mM)
*C*_EN_: concentration of enzyme complex (mM)
*C*_NH_: concentration of 6-APA (mM)
**C**_exp_: experimental concentration (mM)
**C**_est_: estimated concentration (mM)
*K*_m1_: Michaelis–Menten constant for POHPGME consumption (mM)
*K*_m2_: Michaelis–Menten constant for amoxicillin hydrolysis (mM)
*K*_cat1_: catalytic rate constant for POHPGME consumption (mM IU^–1^ min^–1^)
*K*_cat2_: catalytic rate constant for amoxicillin hydrolysis (mM IU^–1^ min^–1^)
*k*_AB_, *k*_AN_, *k*_AOH_, *k*_NH_: inhibition constants of POHPGME, amoxicillin, POHPG and 6-APA, respectively (mM)
*K*_en_: adsorption constant of 6-APA to the enzyme (mM)
*K*_N_, *K*_P_, *K*_S_: equilibrium constants (mM)
*k*_2_, *k*_3_, *k*_4_, *k*_5_, *k*_–4_: reaction rate constants (min^–1^)
*n*: number of experiments
**P**_1_: parameter vector of model 1
**P**_2_: parameter vector of model 2
*S*_1_: first-order Sobol index (dimensionless)
*S*_T_: total-order Sobol index (dimensionless)
*T*_max_: maximum enzymatic conversion (dimensionless)
*v*_AB_: reaction rate of ester consumption (mM min^–1^)
*v*_h2_: reaction rate of amoxicillin hydrolysis (mM min^–1^)
*v*_S_: reaction rate of antibiotic synthesis (mM min^–1^)
ω: relative standard deviation (dimensionless)
**Y**: vector of state variables
