# Supplementary material for: Enzymatic Synthesis of Amoxicillin in a Batch Reactor: Mathematical Modeling, Sensitivity Analysis, and Experimental Validation
Source: ACS Omega. 2025 Jul 18;10(29):31896–907. doi: 10.1021/acsomega.5c03288 (PMC12311728; doi:10.1021/acsomega.5c03288)
Supplement: Supplementary file 1 [file ao5c03288_si_001.pdf]

Supplementary Material for: Enzymatic Synthesis of Amoxicillin in a Batch Reactor: Mathematical Modeling, Sensitivity Analysis, and Experimental Validation

Artur Pedro Martins Neto[1,2,3], Ana Luiza Souza Tavares[1,2,3], Lucas Figueiredo Formigosa[1], Bruno Duarte Gomes[1,2,3], Luciana Rocha Barros Gonçalves[4], and Bruno Marques Viegas[1,5,*]

[1] Faculty of Biotechnology, Federal University of Pará, Belém, PA, 66075-110, Brazil

[2] Laboratory of Neurophysiology Eduardo Oswaldo Cruz, Institute of Biological Science, Federal University of Pará, Belém, PA, 66075-110, Brazil

[3] Simulation and Computational Biology Laboratory, High Performance Computing Center, Federal University of Pará, Belém, PA, 66075-110, Brazil

[4] Department of Chemical Engineering, Federal University of Ceará, Fortaleza, CE, 60455-760, Brazil

[5] Graduate Program in Biotechnology, Federal University of Pará, Belém, PA, 66075-110, Brazil

E-mail addresses

artur.neto@icb.ufpa.br (Artur Pedro Martins Neto), ana.souza.tavares@icb.ufpa.br (Ana Luiza Souza Tavares), lucasfigueiredo1227@gmail.com (Lucas Figueiredo Formigosa), brunodgomes@ufpa.br (Bruno Duarte Gomes), lrg@ufc.br (Luciana Rocha Barros Gonçalves), viegasmbruno@gmail.com (Bruno Marques Viegas).

* Corresponding author.

Mailing address: Graduate Program in Biotechnology, Federal University of Pará, Belém, PA, 66075-110, Brazil.

E-mail address: viegasmbruno@gmail.com (Bruno Marques Viegas).

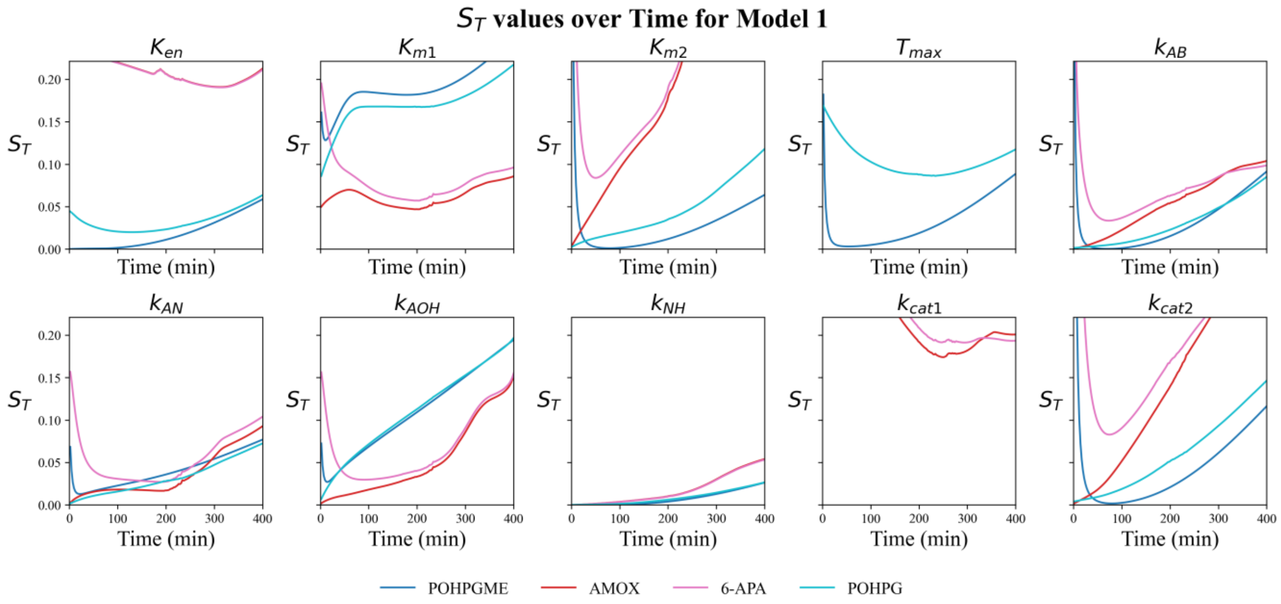

Figure S1 - Temporal profiles of S_T for each kinetic parameter of Model 1, obtained as the average across thirteen experiments. The curves represent POHPGME (dark blue), amoxicillin (red), 6-APA (magenta), and POHPG (cyan).

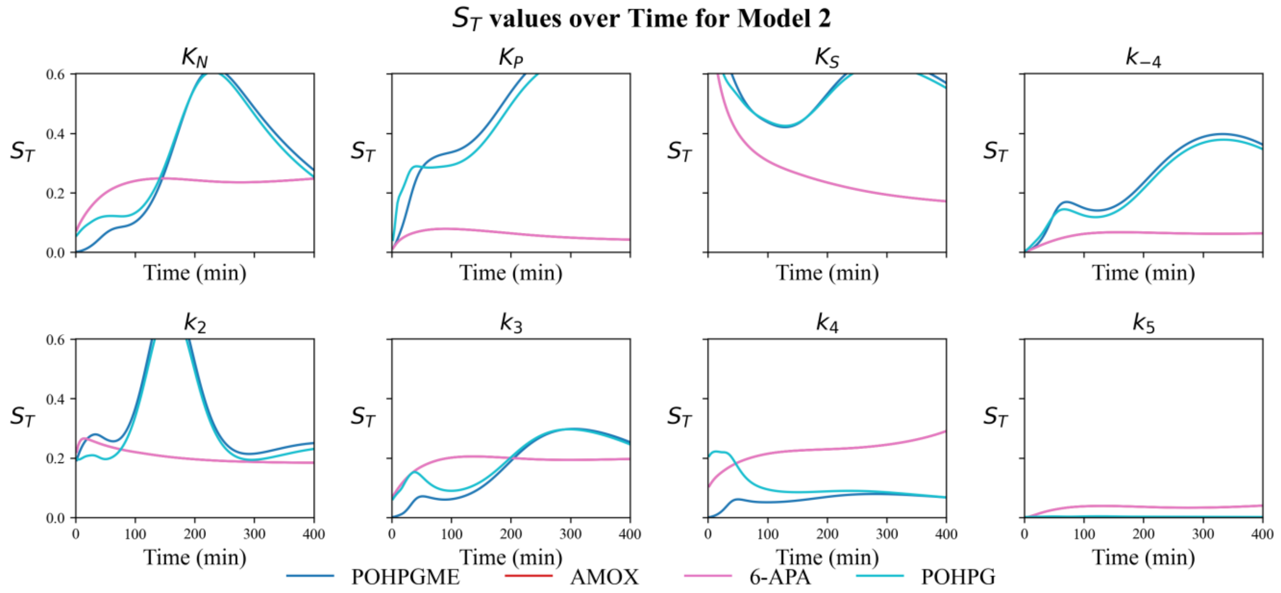

Figure S2 - Temporal profiles of S_T for each kinetic parameter of Model 2, obtained as the average across thirteen experiments. The curves represent POHPGME (dark blue), amoxicillin (red), 6-APA (magenta), and POHPG (cyan).

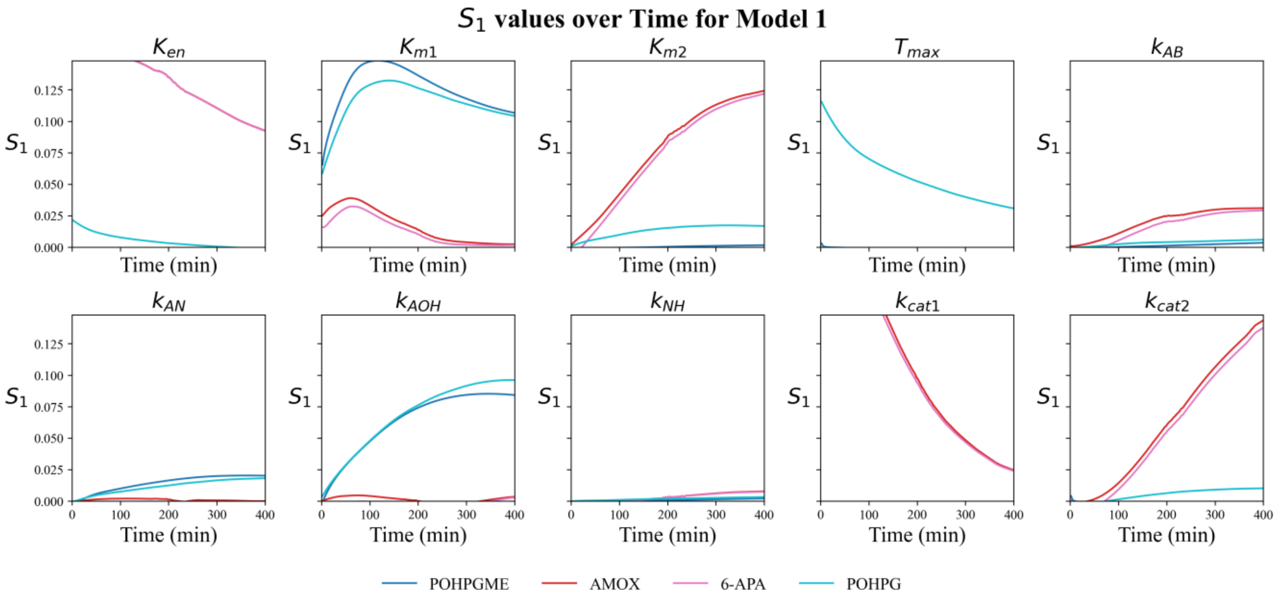

Figure S3 - Temporal profiles of S_1 for each kinetic parameter of Model 1, obtained as the average across thirteen experiments. The curves represent POHPGME (dark blue), amoxicillin (red), 6-APA (magenta), and POHPG (cyan).

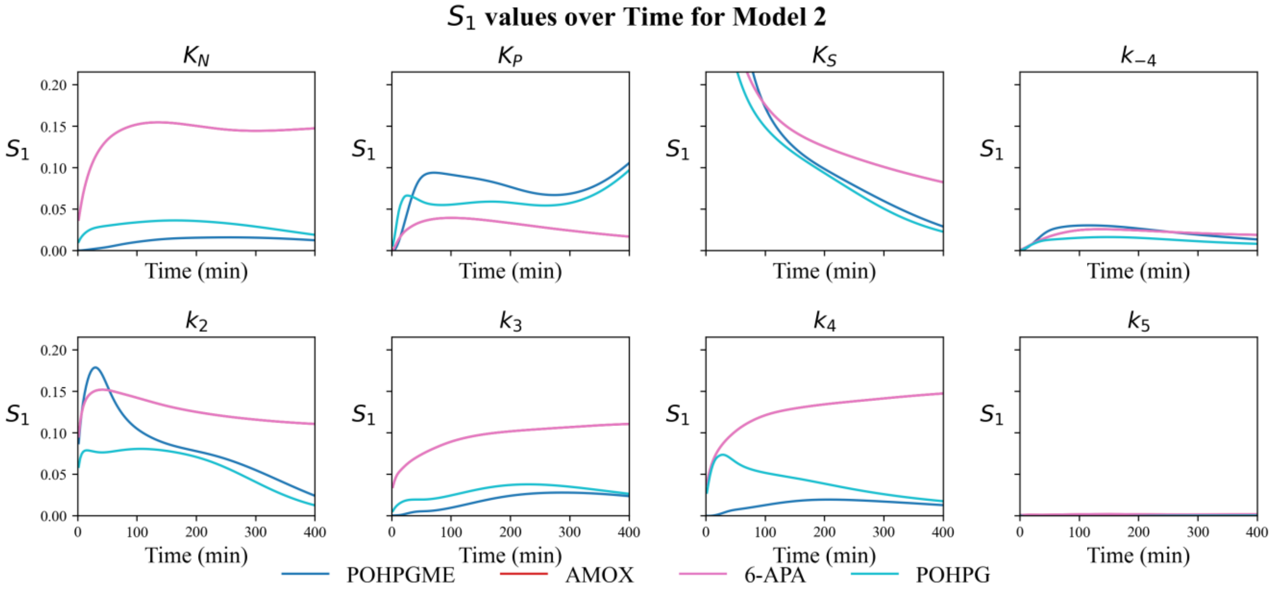

Figure S4 - Temporal profiles of S_1 for each kinetic parameter of Model 2, obtained as the average across thirteen experiments. The curves represent POHPGME (dark blue), amoxicillin (red), 6-APA (magenta), and POHPG (cyan).

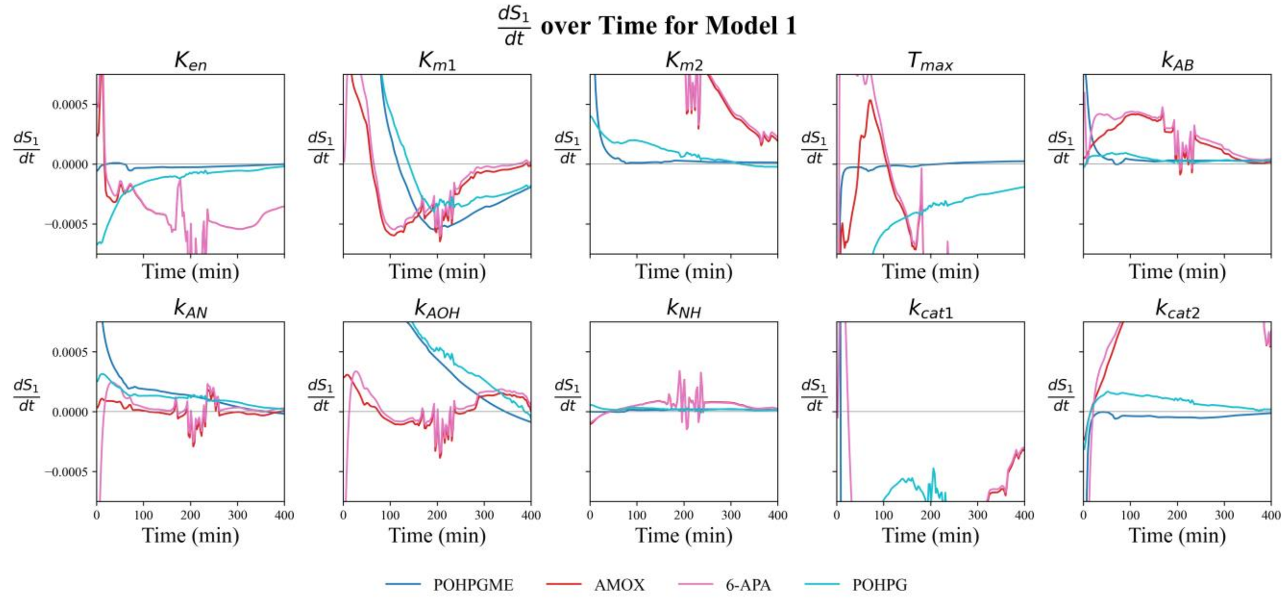

Figure S5 - Temporal profiles of dS_1/dt for each kinetic parameter of Model 1, obtained as the average across thirteen experiments. The curves represent POHPGME (dark blue), amoxicillin (red), 6-APA (magenta), and POHPG (cyan). The gray horizontal line indicates the equilibrium point ($dS_1/dt = 0$).

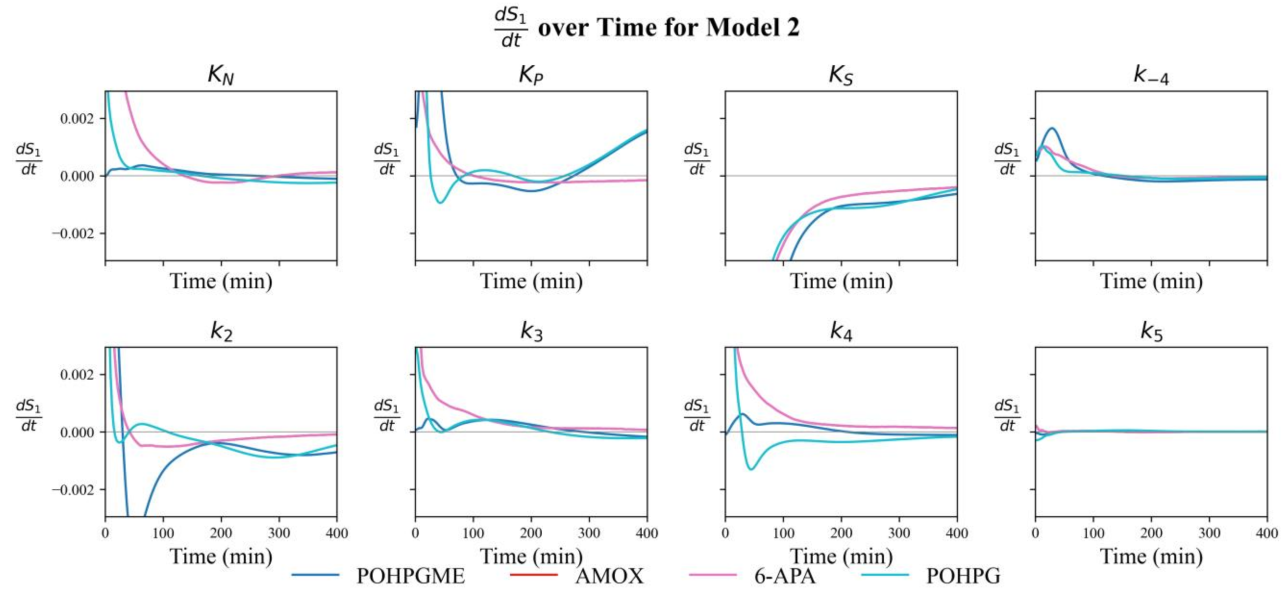

Figure S6 - Temporal profiles of dS_1/dt for each kinetic parameter of Model 2, obtained as the average across thirteen experiments. The curves represent POHPGME (dark blue), amoxicillin (red), 6-APA (magenta), and POHPG (cyan). The gray horizontal line indicates the equilibrium point ($dS_1/dt = 0$).

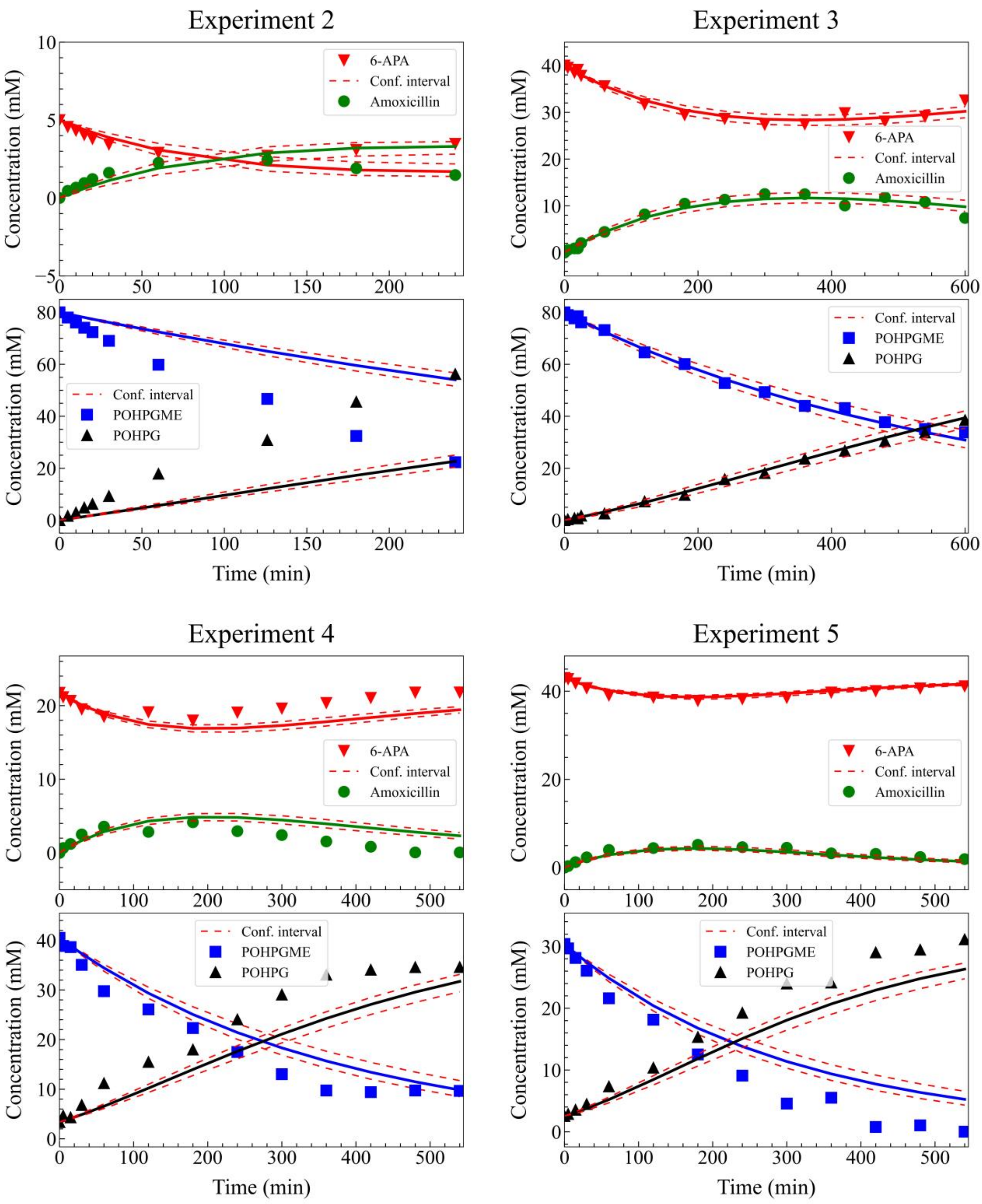

Figure S7 - Experimental data and model predictions for the time course concentrations of 6-APA, POHPGME, amoxicillin, and POHPG in four different experiments (experiments 2, 3, 4 and 5) using Model 1. Symbols represent experimental measurements, while solid lines denote model estimates. Shaded areas or dashed lines indicate the 95% confidence intervals for the model predictions.

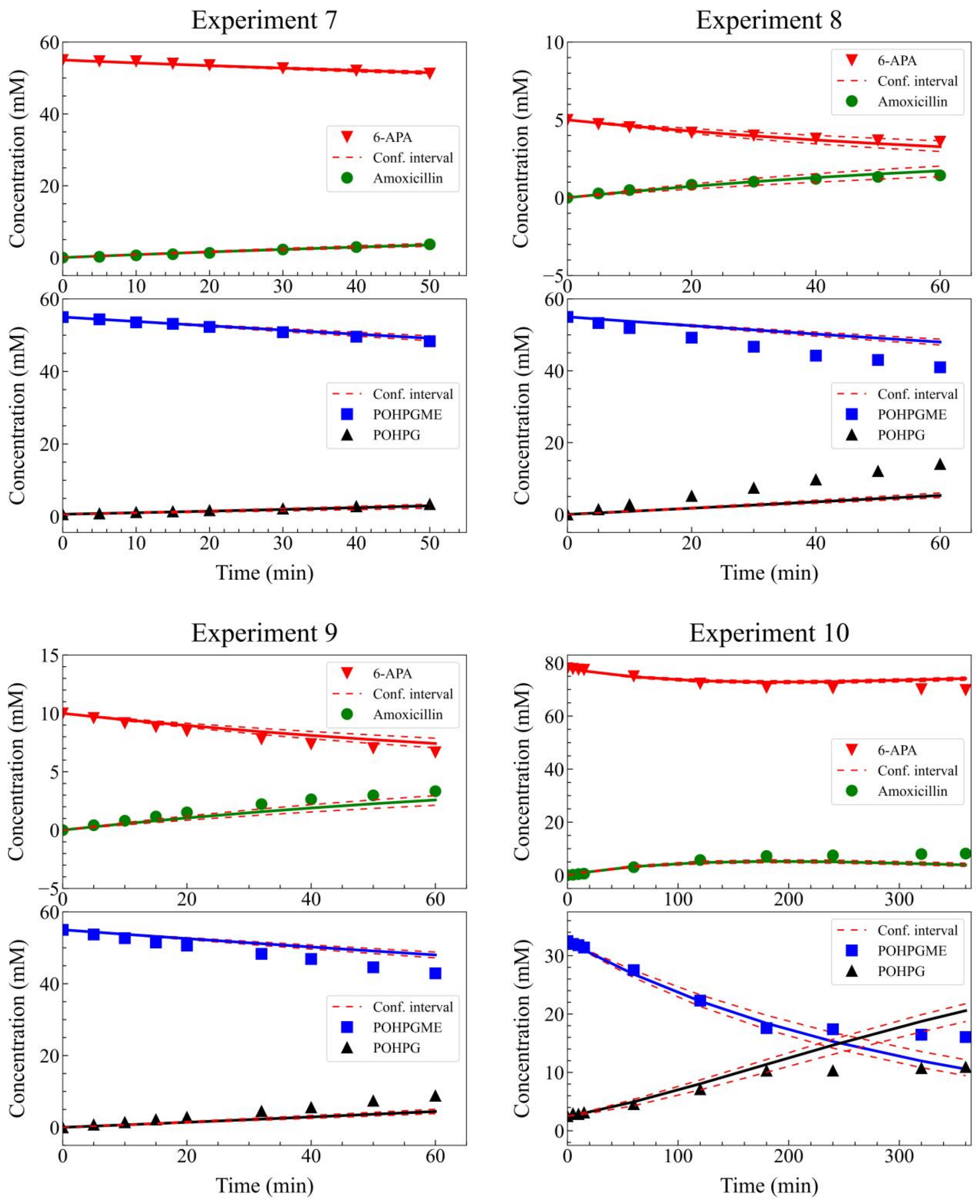

Figure S8 - Experimental data and model predictions for the time course concentrations of 6-APA, POHPGME, amoxicillin, and POHPG in four different experiments (experiments 7, 8, 9 and 10) using Model 1. Symbols represent experimental measurements, while solid lines denote model estimates. Shaded areas or dashed lines indicate the 95% confidence intervals for the model predictions.

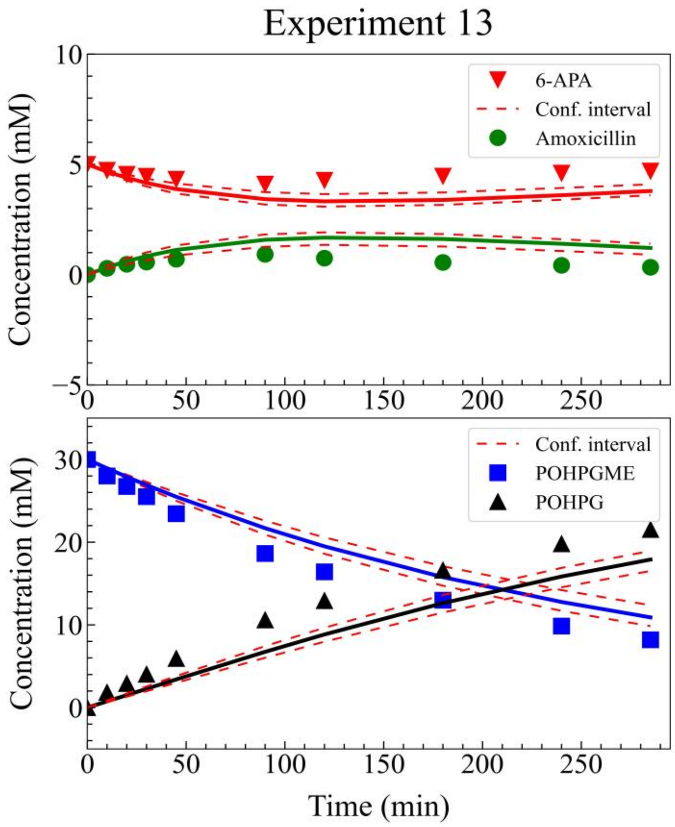

Figure S9 - Experimental data and model predictions for the time course concentrations of 6-APA, POHPGME, amoxicillin, and POHPG in four different experiments (experiment 13) using Model 1. Symbols represent experimental measurements, while solid lines denote model estimates. Shaded areas or dashed lines indicate the 95% confidence intervals for the model predictions.

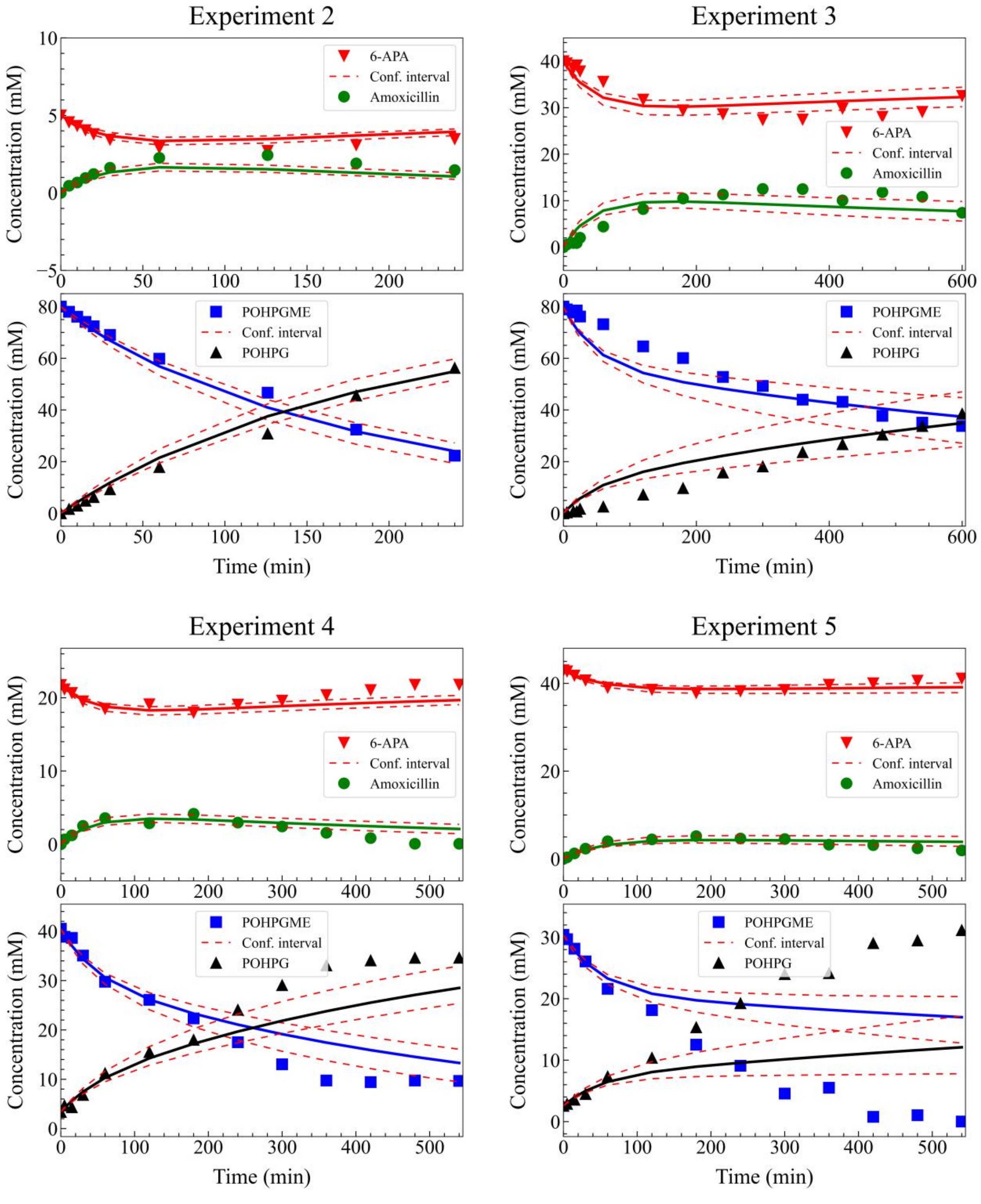

Figure S10 - Experimental data and model predictions for the time course concentrations of 6-APA, POHPGME, amoxicillin, and POHPG in four different experiments (experiments 2, 3, 4 and 5) using Model 2. Symbols represent experimental measurements, while solid lines denote model estimates. Shaded areas or dashed lines indicate the 95% confidence intervals for the model predictions.

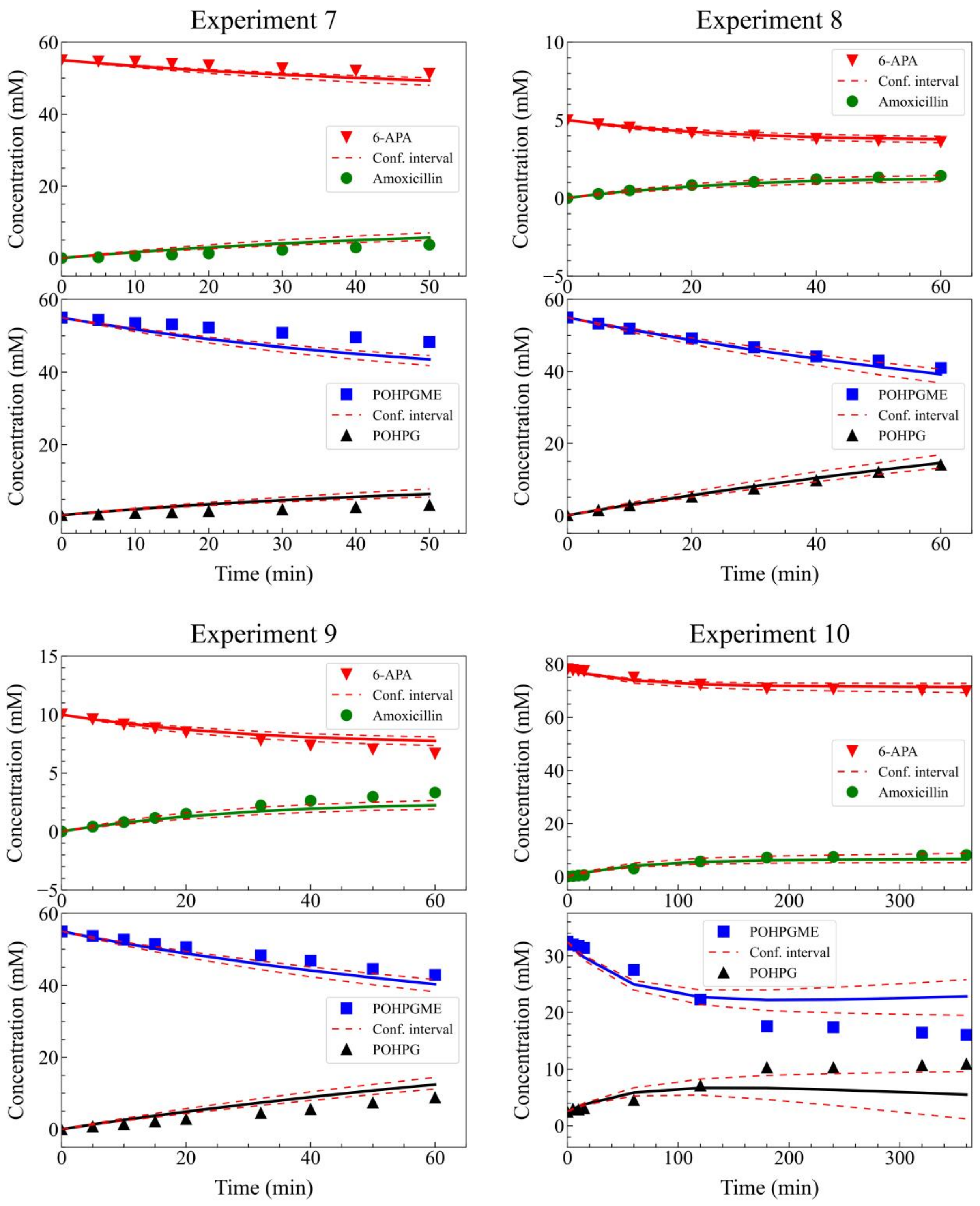

Figure S11 - Experimental data and model predictions for the time course concentrations of 6-APA, POHPGME, amoxicillin, and POHPG in four different experiments (experiments 7, 8, 9 and 10) using Model 2. Symbols represent experimental measurements, while solid lines denote model estimates. Shaded areas or dashed lines indicate the 95% confidence intervals for the model predictions.

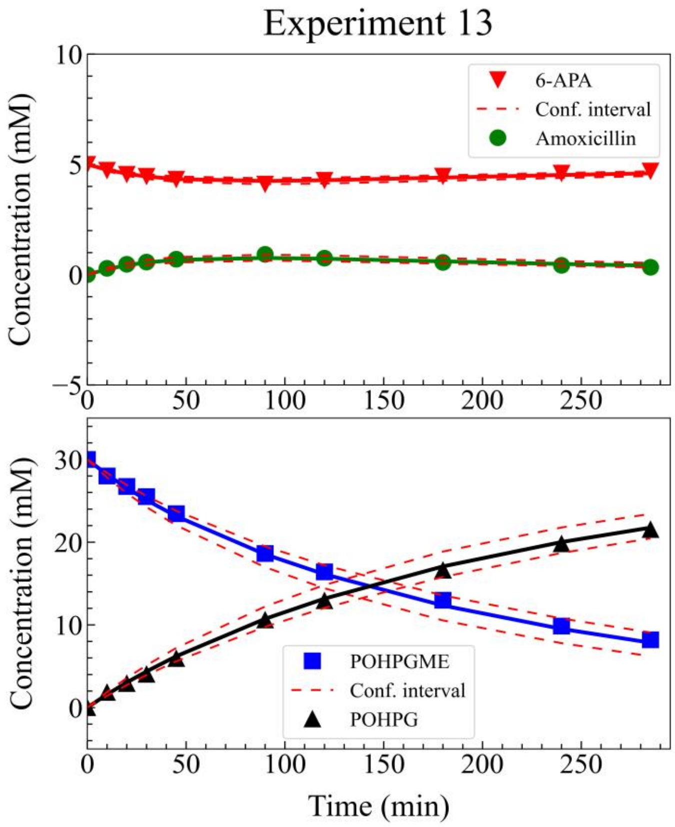

Figure S12 - Experimental data and model predictions for the time course concentrations of 6-APA, POHPGME, amoxicillin, and POHPG in four different experiments (experiment 13) using Model 2. Symbols represent experimental measurements, while solid lines denote model estimates. Shaded areas or dashed lines indicate the 95% confidence intervals for the model predictions.
